# Protocol for *ex vivo* mouse paired single-gonad screening platform for phenotypical characterization

**DOI:** 10.1016/j.xpro.2026.104628

**Published:** 2026-06-10

**Authors:** Martín Andrés Estermann, Karina Flores Rodriguez, James Hunnicutt, Humphrey Hung-Chang Yao

**Affiliations:** 1 Reproductive Developmental Biology Group, National Institute of Environmental Health Sciences, Research Triangle Park, NC 27709, USA; 2 Fabrication and Repair Studio, National Institute of Environmental Health Sciences, Research Triangle Park, NC 27709, USA

**Keywords:** Cell Biology, Cell culture, Cell isolation, Cell-based Assays, Cell separation/fractionation, Developmental biology, Genetics, Metabolism, Microscopy, Model Organisms, Molecular Biology, Stem Cells

## Abstract

Here, we present an *ex vivo* paired single-gonad culture protocol using fetal mouse gonads, optimized for short-term screening with inhibitors, metabolites, or signaling modulators. We describe the experimental design, gonad dissection, paired culture setup, and treatment application to minimize variability. We further outline procedures for downstream phenotypic assessment, including immunostaining, multi-omics analyses, and metabolic measurements of gonadal tissue and culture media.

For complete details on the use and execution of this protocol, please refer to Estermann et al.^1^

## Highlights

• Guidance on 3D printing of resin molds
• Protocol for dissection of fetal mouse gonads and setup for *ex vivo* cultures
• Instructions for analysis by immunostaining, transcriptomics, and lactate measurements

## Abstract (editor highlights)

Publisher’s note: Undertaking any experimental protocol requires adherence to local institutional guidelines for laboratory safety and ethics.

## Graphical abstract

**Figure:**
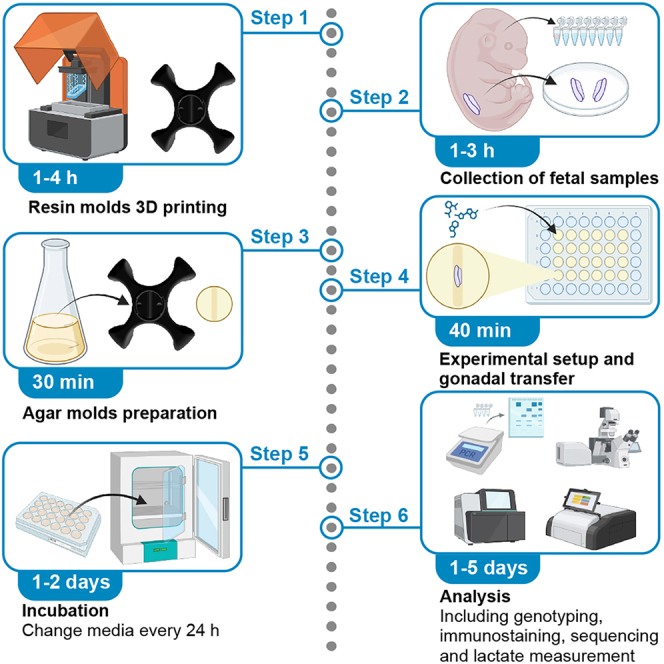

## Before you begin

In mice, gonadal sex differentiation occurs during a narrow developmental window between embryonic day E11.5 and E13.5. During this period, the bipotential gonad undergoes rapid cellular and molecular changes that lead to the establishment of either testis or ovary fate. Because these events occur in utero, direct experimental manipulation and screening during this stage can be technically challenging.

To address these limitations, several ex vivo culture systems have been developed to maintain embryonic gonads outside the embryo while preserving their structure and differentiation capacity.^2^^,^^3^^,^^4^^,^^5^ The current gold-standard methodology for mouse fetal gonadal culture, developed by Capel and Batchvarov,^2^ while widely adopted, this approach cultures multiple gonads within a single agar slab in 10 mm Petri dishes, compromising the individuality of each sample, and requiring substantial amounts of media and reagents. In addition, the original metal molds are difficult to fabricate and typically must be obtained from the originating laboratory, limiting accessibility and reproducibility.

In this protocol, we provide 3D-printable molds optimized for single-gonad culture in standard 48-well plates, along with detailed guidance on resin mold printing, gonad dissection, agar slab preparation, paired culture setup, and treatment application. We also describe downstream phenotypic assessments, including immunostaining, transcriptomic analysis, and metabolic measurements in both tissue and culture media. This protocol is used to investigate the effects of different treatments on fetal gonadal cell differentiation ex vivo. The outlined protocol utilizes E11.5 or E12.5 fetal gonads from wild type C57BL/6 mice (Jackson Laboratory #000664), but the protocol can also be applied to fetal gonads from transgenic mice.

### Innovation

We present an improved and highly accessible system based on a 3D-printed resin mold that can be easily produced in any laboratory. By reducing the size of the agar slabs and culturing paired gonads individually in 48-well plates, our platform preserves biological pairing information, while substantially minimizing reagent use. The paired design reduces the impact of inter-embryo variability, such as differences in developmental stage, litter environment or dissection timing, and allows treatment effects to be assessed primarily from within-embryo differences rather than comparisons between independent samples. This design also facilitates its use as a screening platform for diverse perturbations, including altered culture conditions and metabolic inhibitors, as demonstrated in our recent publication.^1^

### Institutional permissions

All animal procedures were approved by the National Institute of Environmental Health Sciences (NIEHS) Animal Care and Use Committee and are in compliance with a NIEHS-approved animal study proposal (2010-0016). The mice used for this protocol were housed on a 12 h light:dark cycle, temperature range 21–23 °C, relative humidity range from 40 to 50% and ad libitum access to food and water.

### 3D printing of resin molds

**Timing: 3–4 h per mold using a stereolithography (SLA) printer or 1–2 h for 12 molds using a masked stereolithography (MSLA) printer**

1. Requirements:
  a. Personal Protective Elements: eye-protection, respirator/face shield/mask, resin resistant gloves.
  b. UV resin printer (SLA or MSLA).
    ***Note:*** In this protocol we describe the use of the SLA Formlabs Form 3B.
    ***Note:*** We also used the Nexa3D XiP system with optimal results.
  c. Durable type UV resin.
    ***Note:*** We used the Formlabs Rigid 4000.
  d. Sonicator or stirrer washer made for resin printing.
    ***Note:*** We used the Formlabs Form Wash V2.
  e. Isopropyl alcohol at >90% concentration or resin manufacture recommended solvent.
    ***Note:*** We used 99% Isopropyl alcohol.
  f. UV curing station/light box.
    ***Note:*** We used the Formlabs Form Cure V2 120V.
2. Printing instructions:
  a. Load the model (File S1) into the slicer program that you use for your printer.
  b. Arrange the meshes with at least 5 mm between models as loaded on the virtual build plate.
  c. Configure without support material, with the model directly on the build plate, with the flat side down.
    ***Note:*** There is some cupping inside the model, but the volume is so low that it should not present a problem.
  d. Slice the model at the highest vertical resolution/lowest layer height.
  e. Run the print using the gcode provided by the slicer.
    ***Note:*** We recommend printing at least 12 resin molds.
  f. After build plate removal, use either a sonicator or a stir-based parts washer with >90% isopropyl alcohol, or resin manufacturer approved solvent to clean the molds.
    ***Note:*** Be sure that all details are clear of residual resin.
  g. Once cleaned, dry the molds of any excess alcohol using compressed air or allow to air dry.
  h. Place the parts in an ultraviolet curing station for 30–60 min at 405 nm and 60 °C, if equipped with a heater.
    ***Note:*** Further post processing can be applied, like coating, but is not necessary.
    ***Note:*** Do not use molds with rough surfaces, residual resin, distorted grooves, cracks, or incomplete curing.

### Breeding mice

**Timing: 1 h over two consecutive days**

3. Set up several breeding pairs of C57BL/6 mice by placing one female mouse at reproductive age with a single housed male overnight.

***Note:*** We consider females and males in reproductive age between 6 weeks and 6 months of age and between 8 weeks and 10 months of age, respectively.

4. Check plugs the next morning.

***Note:*** We recommend breeding 8–10 females each time. The prescience of a vaginal plug is considered embryonic day 0.5 (E0.5).

***Note:*** As the vaginal plug indicates coitus and not pregnancy, we recommend weighting the plugged females at E0.5 and again at E11.5 or E12.5, as pregnancy is not visibly obvious at these stages. They should gain between 3–6 grams of weight.

### Incubator

5. Tissues are cultured in a standard incubator at 37°C in 5% CO_2_, make sure the incubator is clean and working properly, that CO_2_ is available and that there is sterile autoclaved water in the recipient for humidity control.

### Experimental design

**Timing: 30 min**

6. Prepare an overview of the experimental outline.
  a. Determine the treatment/s that you will test.
    ***Note:*** If an inhibitor will be used, resuspend it in the appropriate vehicle solution, aliquot it and store it properly.
    ***Note:*** We recommend checking in the literature for concentrations used in tissue cultures or organoids and, as a last resource, cells in culture.
    ***Note:*** We typically observe that the concentration needed for gonadal cultures are higher than the ones required for cell cultures. We suggest this to be the starting point and test increasing concentration accordingly.
  b. Determine the minimum number of fetuses required per treatment.
    ***Note:*** The experiment will depend on the number of pregnant mice, and the number of fetuses per pregnant female. We estimate that each pregnant C57BL/6 mice has 6 fetuses. Different treatments or concentrations can be tested depending on the number of fetuses available.
    ***Note:*** As the treatments are done without knowing the sex of the fetus, we recommend allocating 6 fetus/gonadal pairs per treatment (control and vehicle) to increase the chance of having representative tissues from each sex.
  c. Calculate the number of wells/agar slabs required for the culture setup.
    ***Note:*** Two agar slabs/wells will be used per fetus/gonadal pair.
  d. Calculate the amount of culture medium needed for the experimental setup.
    ***Note:*** This protocol uses 180 μl of culture medium per well + 100 μl for the media change per well every 24 h. We estimate 600 μl for pipetting error per gonadal pair/fetus + 10 ml for the agar slabs (see details below).
7. The day of the experiment:
  a. Sanitize the dissection area, stereoscopes and dissecting tools with 70% ethanol.
  b. Sterilize the laminar flow with UV light for at least 20 min.
  c. Sanitize the inside surface area of the laminar flow with 70% ethanol.
    ***Note:*** Make sure to have enough filtered tips, pipettes and sterile Eppendorf tubes available.
  d. Warm up the DMEM/F12 (1:1) media.
    ***Note:*** We use phenol red-free media to image the gonads without needing to remove the media. This protocol works fine with phenol red containing media.
  e. Thaw the heat-inactivated fetal bovine serum (HI-FBS) and 100X Penicillin-Streptomycin (Pen-Strep).
  f. Prepare 50 ml of complete media (10% HI-FBS and 1X Penicillin-Streptomycin) inside the laminar flow hood.
    ***Note:*** Less can be prepared including 10 ml for the agar slabs and 600 μl per gonadal pair for 48 h of culture.
    ***Note:*** Store the remaining complete media at 4°C and use it within a week.
  g. Separate 10 ml of complete media in a 15 ml falcon tube.
  h. Spray the 3D printed resin molds with 70% ethanol and introduce them into the hood.
  i. Add 1ml of cold 1X PBS to the wells of a 24 well plate and place it on an ice bath next to the tissue collection area.
  j. Prepare tubes or a plate for genotyping tissue and place it on an ice bath.

## Key resources table

**Table:** 

REAGENT or RESOURCE	SOURCE	IDENTIFIER
**Antibodies**		

Rabbit Anti-CC3 (1:200)	Cell Signaling	Cat# 9661; RRID: AB_2341188
Mouse Anti-Ki-67 (1:200)	BD	Cat# 550609; RRID: AB_393778
Rabbit Anti-DDX4 (1:200)	Abcam	Cat# ab13840; RRID: AB_443012
Alexa Fluor 488-Anti-Mouse (1:200)	Invitrogen	Cat# A21202; RRID: AB_141607
Alexa Fluor 568-Anti-Rabbit (1:200)	Invitrogen	Cat# A10042; RRID: AB_2534017

**Chemicals, peptides, and recombinant proteins**		

CP 316819 (GPI)	Sigma-Aldrich	Cat# PZ0189
1X phenol red-free DMEM/F12 (1:1)	Gibco	Cat#21041-025
100X Penicillin-Streptomycin (Pen-Strep)	Sigma-Aldrich	Cat# P0781
Heat-inactivated Fetal Bovine Serum (HI-FBS)	Gibco	Cat#16140071
ReadyMix REDTaq	Sigma-Aldrich	Cat#R2648
Bacto Agar	BD	Cat#214010
Sucrose	Sigma-Aldrich	Cat#S5016
GelRed Prestain Plus 6X DNA Loading Dye	Biotium	Cat#41011
Trackit 1kb plus DNA ladder	ThermoFisher Scientific	Cat#10488085
Agarose	Millipore-Sigma	Cat#A9539
O.C.T. Compound	Tissue-Tek	Cat#4583
Antigen Unmasking Solution, Citric Acid Based (100X)	Vector Laboratories	Cat#H-3300
Tris Base (Trizma)	Sigma-Aldrich	Cat#T1503
Boric Acid	Sigma-Aldrich	Cat#B0394
EDTA	Sigma-Aldrich	Cat#E9884
NaOH	Fisher scientific	Cat#BP359
Benzyl alcohol	Sigma-Aldrich	Cat#108006
Benzyl benzoate	Sigma-Aldrich	Cat#B6630
HCl	Sigma-Aldrich	Cat#320331
Triton X-100	Fisher scientific	Cat#BP151-100
Normal Donkey Serum	Sigma-Aldrich	Cat# S30-100ML

**Critical commercial assays**		

Arcturus™ PicoPure™ RNA Isolation Kit	Applied Biosystems	Cat# KIT0204
RNase-Free DNase Set	Qiagen	Cat# 79254
Qubit™ RNA High Sensitivity kit	ThermoFisher Scientific	Cat# Q32852
Tecan’s Ovation® RNA-Seq System V2	Tecan	Cat# 7102
Tecan’s Celero™ EZ DNA-Seq	Tecan	Cat# 30188860
Lactate-Glo Assay	Promega	Cat# J5021
Qubit Protein Assay	ThermoFisher Scientific	Cat# Q33211
TrueView Autofluorescence Quenching Kit	Vector Labs	Cat#SP-8400
ProLong™ Diamond Antifade Mountant	Invitrogen	Cat#P36970
Antigen Unmasking Solution, Citric Acid Based	Vector Labs	Cat#H-3300-250
High Sensitivity RNA ScreenTape + Sample Buffer	Agilent	Cat# 5067-5578/80

**Deposited data**		

GPI RNA-seq	Estermann et al.^1^	GEO: GSE293017

**Experimental models: Organisms/strains**		

C57BL/6 mice. Females between 6 weeks and 6 months of age. Males between 8 weeks and 10 months of age.	Jackson Laboratory	000664

**Oligonucleotides**		

Sx Fw: 5′-GATGATTTGAGTGGAAATGTGAGGTA-3′	IDT	https://doi.org/10.1159/000348677
Sx Rv: 5′-CTTATGTTTATAGGCATGCACCATGTA-3′	IDT	https://doi.org/10.1159/000348677

**Software and algorithms**		

ImageJ/FIJI	Schindelin et al.^6^	RRID:SCR_003070
Prism	GraphPad Software	RRID:SCR_002798

**Other**		

Biological Safety Cabinets	Nuaire	N/A
Forma Steri-Cycle CO2 Incubators	ThermoFisher Scientific	N/A
Stereo Microscope	Leica	MZ16
Super thin tip forcep	Dumont	Cat#0103-5-PO
Sterile 24 well plate, flat bottom	ThermoFisher Scientific	Cat#142475
Sterile 48 well plate, flat bottom	ThermoFisher Scientific	Cat#152640
Sterile 96 well plate, flat bottom	Millipore-Sigma	Cat#CLS3795
White 96 well plate, flat bottom	ThermoFisher Scientific	Cat#136101
HD Camera	Leica	DMC6200
GloMax Discoverer microplate reader	Promega	GM3000
Tissue-Tek Cryomold 10 x 10 x 5 mm	Sakura	Cat#4565
Superfrost Plus Microscope Slides	ThermoFisher Scientific	Cat#1255015
Cryostat	Leica	CM3050 S
Confocal Laser Scanning Microscope	Zeiss	LSM900
ChemiDoc Imaging System	Bio-Rad	N/A
Thermal Cycler	Bio-Rad	C1000
Sequenza™ Immunostaining Center Slide Rack	Epredia	Cat#73310017
Disposable Immunostaining Chamber	Epredia	Cat#72110017
Gel Electrophoresis system	ThermoFisher Scientific	N/A
Form 3 B 3D printer	Formlabs	N/A
Rigid 4000 Resin	Formlabs	N/A
Form Wash V2	Formlabs	FH-WA-03
Form Cure V2 120 V	Formlabs	FH-CU-120V-02

## Materials and equipment

**Table:** Complete media

Reagent	Final concentration	Amount for 50 ml	Amount for 10 ml	Amount per gonadal pair
HI-FBS	10%	5 ml	1 ml	6 μl
100X Pen-Strep	1X	0.5 ml	0.1ml	60 μl
DMEM/F12 (1:1)	N/A	44.5 ml	8.9 ml	534 μl
Total	N/A	50 ml	10 ml	600 μl

Note: Store at 4°C for 1 week.

**Table:** Sucrose 30%

Reagent	Final concentration	Amount
Sucrose	30%	30 g
PBS 1X	N/A	70 ml
Total	N/A	100 ml

Note: Store at 4°C for up to 1 month.

**Table:** 10X TBE

Reagent	Final concentration	Amount
Tris Base	0.89 M	108 g
Boric Acid	0.89 M	55 g
0.5 M EDTA	20mM	40 ml
Water	N/A	800-900 ml
Total	N/A	1000 ml

***Note:*** To make 1X TBE dilute 1:10 in water.

***Note:*** Store at room temperature (20–23°C) for up to 6 months.

**Table:** 1M Tris-HCl pH8

Reagent	Final concentration	Amount
Tris Base	1 M	121.1 g
Water	N/A	Bring up to 1000 ml
Total	N/A	1000 ml

***Note:*** We recommend dissolving the Tris Base completely into 800 ml of distilled water and then measure the pH. Adjust with 1M HCl until pH=8 and bring up to 1000 ml volume.

***Note:*** Store at room temperature for up to 6 months.

**Table:** 1M Tris Base (Trisma)

Reagent	Final concentration	Amount
Tris Base	1 M	121.1 g
Water	N/A	Bring up to 1000 ml
Total	N/A	1000 ml

***Note:*** Do not adjust pH.

***Note:*** Store at room temperature for up to 6 months.

**Table:** 50mM Tris pH7.5

Reagent	Final concentration	Amount
1 M Tris Base	50mM	5 ml
Water	N/A	95 ml
Total	N/A	100 ml

***Note:*** Adjust pH to 7.5 with 1M HCl.

***Note:*** Store at room temperature for up to 6 months.

**Table:** 50mM NaOH

Reagent	Final concentration	Amount
NaOH	50mM	200 mg
Water	N/A	Fill up to 100 ml
Total	N/A	100 ml

***Note:*** Store at room temperature for up to 6 months.

**Table:** Blocking solution immunofluorescence (cryosections)

Reagent	Final concentration	Amount
Donkey Serum	5%	2.5 ml
Triton X-100	0.1%	50 μl
PBS 1X	N/A	49.5 ml
Total	N/A	50 ml

***Note:*** Store aliquots at −20°C for up to 6 months, avoid repeated freeze-thaw cycles.

**Table:** Blocking solution immunofluorescence (whole mount)

Reagent	Final concentration	Amount
Donkey Serum	10%	5 ml
Triton X-100	1%	500 μl
PBS 1X	N/A	44.5 ml
Total	N/A	50 ml

***Note:*** Store aliquots at −20°C for up to 6 months, avoid repeated freeze-thaw cycles.

**Table:** BABB

Reagent	Final concentration	Amount
Benzyl alcohol	33.3%	33.3 ml
Benzyl benzoate	66.7%	66.7 ml
Total	N/A	100 ml

***Note:*** Store at room temperature, protected from light, for up to 6 months.

**Table:** 0.6M HCl

Reagent	Final concentration	Amount
HCl 37% w/w	0.6 M	5 ml
Water	N/A	95 ml
Total	N/A	100 ml

***Note:*** Store at room temperature for up to 6 months.

***Note:*** Always add acid to water.

## Step-by-step method details

This protocol is a modified version of the agar slab gonad culture method by Capel and Batchvarov.^2^

### Collection of fetal mouse gonads

**Timing: 1**–**3 h**

This section details how to isolate gonad-mesonephros complexes from E11.5 or E12.5 mouse fetuses.

1. Euthanize E11.5 or E12.5 pregnant mice using CO_2_ asphyxiation followed by an approved secondary method such as cervical dislocation.

***Note:*** If several pregnant mice will be dissected, do it one at the time to minimize cell death.

2. Collect the fetuses from the uterus and place them in a glass petri dish containing cold 1X PBS on ice.
3. Manually dissect the gonadal-mesonephros complexes in glass petri dish containing 1X PBS under a stereoscope (Methods video S1).
  ***Note:*** Two micro dissection forceps (tip dimension 0.06 x 0.1 mm) are most useful for removing fetal gonads. A pair 1 ml syringes with 30-gauge needles attached are used to separate and clean the gonads.
  a. Remove the head of the fetus and place it in a 96 well plate or strips for later sex genotyping.
    ***Note:*** Make sure to give every embryo an identification number and label the samples properly to match the sex to the gonads.
  b. Grab the embryo under the limb buds, place the forceps in an angle and remove the tissue above the limb buds.
  c. Pin the embryo down with forceps, dorsal side down using your non dominant hand.
  d. With the other forceps remove the remaining primitive gut tissue to expose the urogenital system located in the back of the embryo.
  e. Place the forceps under the urogenital system and move them anteriorly and posteriorly to release it from the body, gently avoiding destroying them.
  f. Pinch the caudal end of the gonad and lift it up, separating the tissues from the body.
  g. With the needles, carefully separate the gonads-mesonephros complexes from the connective tissue and mesentery by cutting along the edge of the gonad with the needle.
  h. Trim any additional tissues without damaging the gonad/mesonephros.
  Methods video S1. Isolation of gonad/mesonephros complexes from an E11.5 embryo, related to step 3
4. Transfer each gonadal pair to the 24 well plate on ice containing 1 ml of cold PBS per well using a p200 pipette.
5. Repeat for all fetuses until all gonads are collected.
6. Repeat until all pregnant mice are collected.

***Note:*** Discard the gonadal pair if one of the gonads is damaged during dissection or transfer.

### Preparation of agar slabs

**Timing: 30 min**

This section describes how to generate agar slabs using the 3D-printed resin molds.

7. Measure 1.5 mg of bactoagar in a 20 ml Pyrex Erlenmeyer and add 10 ml of complete media. Cover the top of the flask with film.
8. Heat 40 ml of deionized and filtered water for 1 minute in a 100 ml beaker using a microwave or until it boils (bubbles visible). Set aside.

***Note:*** Careful when handling as it will be boiling hot!

9. Heat the Erlenmayer with the agar media mixture for short intervals in the microwave.
10. Stop when bubbles are forming, swirl the solution to remove the bubbles and continue heating until the agar is completely dissolved.

***Note:*** Be careful with the bubbles forming as it can overflow the Erlenmayer.

***Note:*** Look through the flask to see if the agar is dissolved. Pay attention to the bubbles size, they will become larger when agar is dissolved. Small white particles can remain in the solution due to the serum proteins being denaturalized.

11. Place the hot Erlenmeyer into the beaker with hot water.
12. Spray with ethanol and place it in the hood.
13. Add 90 μl of the agar mixture into each resin mold, creating a dome structure, careful not to include bubbles (Methods video S2).

***Note:*** 10 ml of agar solution is enough for 36 wells.

***Note:*** We recommend having at least 12 resin molds or more to generate several agar slabs at the same time.

14. Wen solidified, remove the agar slabs with a mini spatula and place them inside the wells of a 48 well plate with the dome side down and the groove side up (Methods video S3).

***Note:*** As time passes the agar will solidify faster. The beaker water bath will make the agar stay liquid for longer. If the agar solidifies completely in the Erlenmeyer, reheat it in microwave for short intervals until it is liquid again.

15. Repeat until all wells are filled (1 gonad per well), 2 wells per gonadal pair (fetus).

***Note:*** Use the inner wells of the 48-well plate whenever possible and avoid edge wells because they are more prone to evaporation. In total we fit 12 gonadal pairs/fetus per plate.

Methods video S2. Pipetting of agar solution into the resin molds to generate agar slabs, related to step 13

Methods video S3. Removal of agar slabs from resin molds and transfer to culture wells, related to step 14

### Experimental setup

**Timing: 20 min**

This section describes how to set up the experimental plate for single gonadal cultures.

16. Determine what you are trying to evaluate. In this case, as an example we are testing the effect of the glycogen phosphorylase inhibitor (GPI), but this culture method can be used for different treatments.

***Note:*** We have successfully tested different inhibitors, media compositions, media supplementations and culture conditions. Examples can be found in our recent publication.^1^

17. Each well requires 180 μl of media.

***Note:*** For easy calculation and to account for pipetting error make 200 μl of media per well.

18. Dilute your inhibitor to the desired concentration and include the corresponding vehicle solution controls.

***Note:*** We recommend starting by testing different inhibitor concentrations using multiple gonadal pairs per treatment (optimal 6), as the analysis will be performed blindly without knowing the sex of the fetus.

19. Add 180 μl of the corresponding media to each well containing an agar slab.

***Note:*** We recommend creating a row of gonads used for controls and the corresponding gonad pair in a row of inhibitors per sample. Comparisons will be done between gonads from the same pair, one in vehicle control and the other in the inhibitor.

20. Add 500 μl of sterile 1X PBS on each of the unused border wells to minimize water evaporation from the inner culture wells.

### Gonadal transfer onto the agar slabs

**Timing: 20 min**

This section details how to transfer the gonads inside the grooves of the agar slabs.

21. As this occurs outside of the laminar flow:
  a. Sanitize the station working station by spraying 70% ethanol.
  b. Sterilize forceps and 20 μl pipette with filtered tips.
22. Use the pipette to transfer one of the gonads from one pair into a well (vehicle) and the other one from the same pair into the treatment well, in this case GPI.

***Note:*** Attempt to place it on the groove of the agar slab. If necessary, this can be done under a stereoscope.

***Note:*** Try to transfer a little PBS as possible when transferring the gonads. We recommend setting the pipette to 10 μl.

23. Once all gonads are transferred, assess them under a stereoscope.

**Figure 1:**
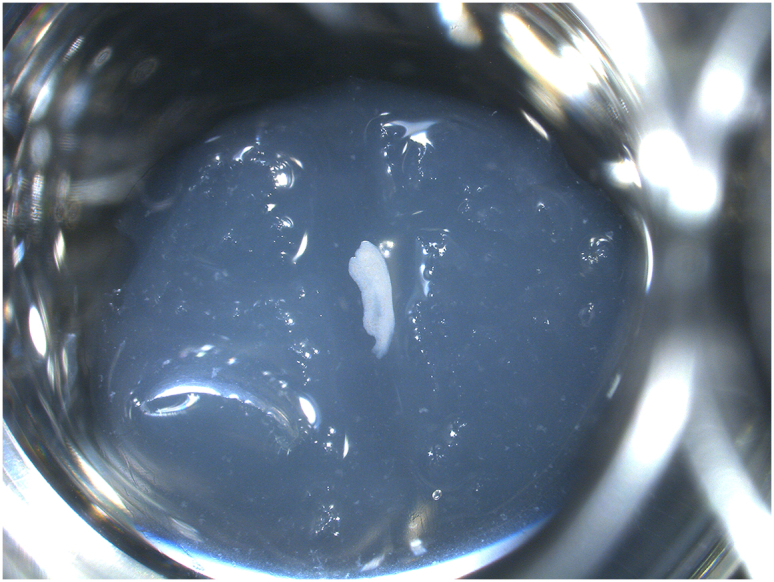
Stereomicroscope image of an E12.5 gonad correctly positioned in the groove of the agar slab

***Note:*** If any gonad is not properly located in the agar slab groove (Figure 1), use the sterilized forceps to orient them.

***Note:*** To avoid breaking the gonad, grab it from the mesonephric side at the posterior end or use the forceps to push them gently into the groove.

24. Close the plate lid and place it in the incubator for at least 24 h.

***Note:*** We typically culture them for 48 h.

### Media change

**Timing: 30 min**

This section details how to refresh the media without perturbing the gonad.

We replace 100 μl of media every 24 h, to restore the inhibitor and refresh the media.

25. Prepare the complete media with fresh inhibitors and vehicle solutions.

***Note:*** We use the complete media prepared from the day before, stored at 4 °C. We add fresh inhibitors and vehicle solutions to these media.

26. Sanitize the working area including a stereoscope.
27. Open the plate and under the scope aspirate 100 μl on one side of the agar slab, with a 200 μl pipette with a filtered tip.

***Note:*** Pipette gently to avoid moving the gonads from the groove.

28. Close the plate lid, transfer the plate into the hood.
29. In the hood, add fresh 100 μl of media.
30. Close the plate and place it back in the incubator.

***Note:*** The 100 μl of media removed from the well in step 2 can be saved for further analysis by transferring it into a 96 well plate. Seal the plate with parafilm and store −20 °C.

***Note:*** Check media volume and tissue position before and after media changes. If substantial evaporation is observed exclude the affected wells.

### Sex genotyping

**Timing: 6 h**

**Timing: 2 h 10 min (step 31)**

**Timing: 30 min (step 32)**

**Timing: 1 h 40 min (step 33)**

**Timing: 30 min (step 34)**

**Timing: 50 min (step 35)**

In this section we explain how to perform sex genotyping by PCR^7^ using the fetal head tissue collected previously as template.

31. Digestion:
  a. Add 100 μl of 50 mM NaOH to each well with fetal head tissue.
  b. Incubate for 2 h at 98 °C in a thermocycler.
  c. Stop reaction with 50 μl 1 M Tris-HCl pH8.
  d. Store at 4 °C.
32. PCR set up:

***Note:*** This protocol uses a 2x ReadyMix REDTaq that contains everything needed for PCR except the primers and includes the gel loading buffer.

***Note:*** If you are using other Taq or PCR mix, convert it to your usual genotyping protocol.

**Table:** 

Reagent	Amount
Tissue digestion	2 μl
2x ReadyMix REDTaq	12.5 μl
Sx Fw Primer 20 μM	0.625 μl
Sx Rv Primer 20 μM	0.625 μl
ddH_2_O	9.25 μl

***Note:*** Multiply the volumes (except the tissue digestion) by the number of samples you are going to genotype. We recommend adding an extra sample every 10 tubes to account for pipetting error.

33. PCR Run and cycling conditions:

***Note:*** This PCR program is optimized for the REDTaq.

**Table:** 

Steps	Temperature	Time	Cycles
Initial Denaturation	94°C	3 min	1
Denaturation	94°C	30 sec	35 cycles
Annealing	60°C	35 sec	–
Extension	72°C	60 sec	–
Final extension	72°C	5 min	1
Hold	4°C	forever

***Note:*** Extension temperature depends on type of Taq enzyme used (REDTaq: 72 °C). Adjust to your polymerase temperature.

**Figure 2:**
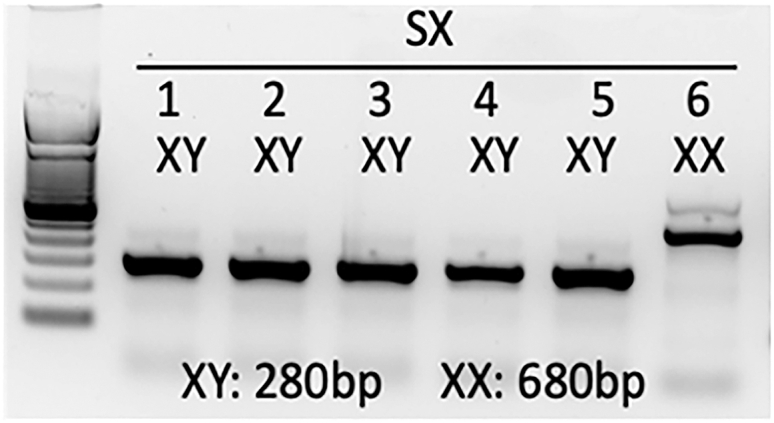
Representative genotyping PCR results and agarose gel electrophoresis showing XX and XY samples
  b. Record the XX or XY result together with the unique embryo identification number used during dissection and culture.
  c. Link the sex genotype to both contralateral gonads from the same embryo, including the control and the matched treated gonad.
  d. Maintain the embryo ID, sex, treatment condition, and pairing information throughout downstream processing.
    ***Note:*** Since fetal gonadal development is sexually dimorphic, XX and XY samples should be analyzed separately. Sex can be included as a biological variable and treatment-by-sex interactions must be considered.

34. Agarose gel making.
  ***Note:*** This recipe is for a 1.5% agarose gel, which will work for the expected band size for both male and female mice.
  a. Prepare 1X TBE buffer (Tris-Borate-EDTA).
  b. Dissolve 2.25 g agarose in 150 ml 1X TBE buffer in an Erlenmeyer.
  c. Heat in microwave to dissolve agarose, regularly stop to mix and make sure it does not overflow.
  d. Let the solution cool down in a water bath until is safe to the touch.
    ***Note:*** Nucleic acid gel stain can be added to the warm agarose here. We will add the stain to each individual sample.
  e. Prepare the gel rack and position the combs.
  f. Pour warm gel in gel rack, make sure there is not bubble particularly around the combs.
  g. Let it gellify completely.
35. PCR gel migration:
  a. Move the agarose gel into the gel box, fill the gel box with 1X TBE buffer and remove the combs.
  b. Once the PCR is done, separate 10 μl of the sample into new tubes and add the nucleic acid gel stain to each sample.
    ***Note:*** We use a multichannel pipette to transfer the sample into new tubes. As we use a 6X GelRed Prestain Plus DNA Loading Dye, we add 2 μl to each sample.
  c. Load 10–12 μl of the PCR + Gel red mixture into the gel wells, using a multichannel pipette.
    ***Note:*** Store the remaining PCR products in the fridge, as they can be used to re-run inconclusive samples.
  d. Mix 4 μl of the ladder and 1 μl of the Gel red and load the mixture into 1 well.
  e. Run gel for 30 min at 100 V or more if necessary.
  f. Image in a gel imaging system.
36. Record genotyping results and assign sex to each gonadal pair:
  a. Identify the sex of each embryo based on the PCR band pattern.
    ***Note:*** Using these primers, XY samples have a single 280bp band and XX samples a single 680bp band (Figure 2).

### Gonadal processing after culture

**Timing: 20 min**

This section describes how to retrieve the gonads after the culture, for subsequential analysis.

37. Remove the plate from the incubator.
38. Push the gonad out gently from the agar slab using forceps, without damaging the gonad.
39. Remove the agar slab using the back of a forceps.

***Note:*** Paying attention not to drag the gonad with the agar slab by mistake.

40. Image each individual gonad using a stereoscope camera.
41. Proceed with the appropriate sample processing depending on the technique being used for analysis. We provide some examples below.

***Note:*** The remaining media left in the well after culture can be saved for further analysis by transferring it into a 96 well plate. Seal the plate with parafilm and store at −20 °C.

### Cryosectioning and immunostaining

**Timing: 3 days**

**Timing: 30 min (step 42)**

**Timing: 2 days (step 43)**

This section describes how to process the gonadal samples for immunostaining in cryosections.

We suggest starting the characterization using immunostaining in gonadal sections as this technique allows the phenotypical characterization using several markers including apoptosis to evaluate inhibitor cytotoxicity.

42. Sample processing:
  a. Remove the culture media and replace it with 500 μl of freshly prepared 4% PFA in 1X PBS.
  b. Fix overnight (16–18 h) at 4 °C or 1 hour at room temperature.
  c. Remove the fixative and perform three 10 min washes with 1X PBS at room temperature.
  d. Replace the 1X PBS with 30% sucrose in 1X PBS and incubate them overnight at 4°C.
  e. Transfer the samples into the cryo-molds (10 mm x 10 mm x 5 mm) filled with O.C.T. compound.
    ***Note:*** Use a needle to push them to the bottom and place them in the desired orientation.
    ***Note:*** As we know the sex of the samples by now, we typically create O.C.T. blocks of same sex controls and inhibitors, in different rows (Figure 3). To know the orientation and identify the samples after sectioning, we paint with a marker the right-top corner of the cryo-mold. This staining is transferred to the O.C.T. block but does not affect the staining.

**Figure 3:**
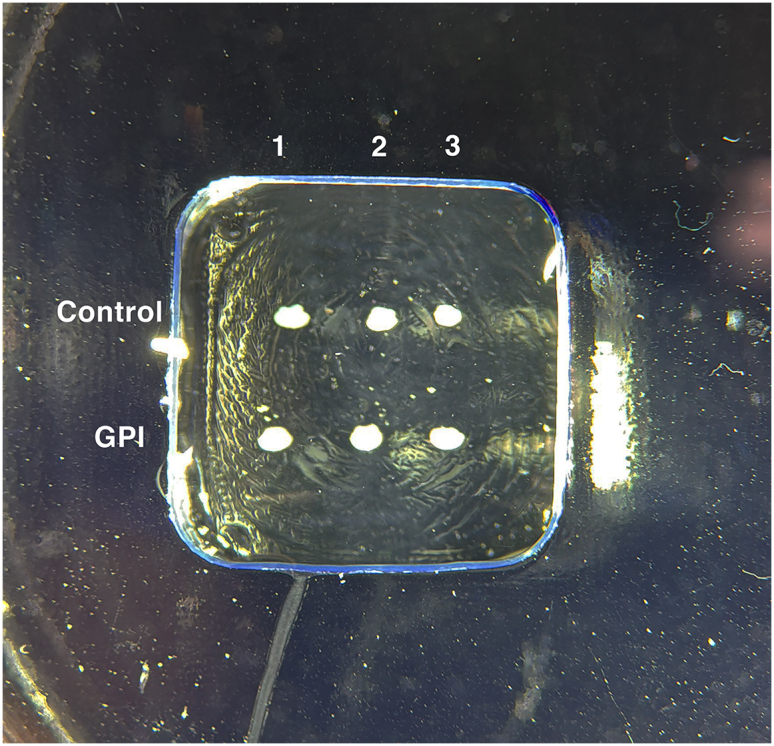
Example of sample placement in cryo molds, including three matched control and GPI-treated gonads
  f. Transfer the O.C.T blocks in the cryo-molds to a −80 °C freezer.
43. Cryosectioning and immunostaining
  a. Transfer the O.C.T blocks from the −80 °C to the cryostat and let them equilibrate.
  b. Section 10 μm thick samples using a cryostat at −20°C.
    ***Note:*** We collect all consecutive sections in 4-5 different positively-charged glass slides alternating in between slides.
    ***Note:*** Remember and annotate the blue corner orientation when sectioning and how it transfers into the glass slide. This is crucial to identify the samples when imaging.
  c. Perform antigen retrieval in the cryosections using pre-warmed citrate-based antigen retrieval solution.
    ***Note:*** We microwave the slides in the 1X pre-warmed citrate-based antigen retrieval solution (unmasking solution, citric acid based, Vector laboratories) at 10% power for 20 min and let cool down for 30 min at room temperature.
    ***Note:*** This can be avoided if not necessary. It will depend on the antibodies ability to detect antigens.
  d. Remove the antigen retrieval solution and replace it with 1X PBS at room temperature.
  e. Transfer the slides to the Sequenza manual immunohistochemistry system, using 500 μl of PBS.
    ***Note:*** Immunofluorescence can be done in staining trays if necessary. We use the Sequenza immunohistochemistry system as it reduces the volumes used. We reuse the disposable immunostaining chamber if they don’t have scratches.
  f. Block and permeabilized the samples in 0.1% triton X-100 in 1X PBS with 5% normal donkey serum for 1 hour at room temperature.
  g. Incubate the samples with the desired primary antibodies diluted in blocking buffer overnight at 4 °C.
  h. Wash the samples three times using 0.1% triton X-100 in 1X PBS.
  i. Incubate samples with secondary antibodies diluted in blocking buffer for 1 hour at room temperature.
  j. Wash the slides 1 time with 0.1% triton X-100 in 1X PBS and 2 times with 1X PBS.
    ***Note:*** If needed, autofluorescence can be quenched. We suggest this for testicular samples as Leydig cells tend to be autofluorescent. We use the TrueView Autofluorescence Quenching Kit for 3 min then wash the samples for 10 min using 500 μl of 1X PBS.
  k. Counterstain samples with DAPI and mount them.
    ***Note:*** We use ProLong™ Diamond Antifade Mountant for mounting and we let them harden for at least overnight.
  l. Image the samples next using confocal microscope.
    ***Note:*** As an example, we are showing the staining for proliferation (Ki-67) and apoptosis (cleaved caspase 3) in XY gonads cultured with GPI (75 or 100 μM) or vehicle solutions (Figure 4).

**Figure 4:**
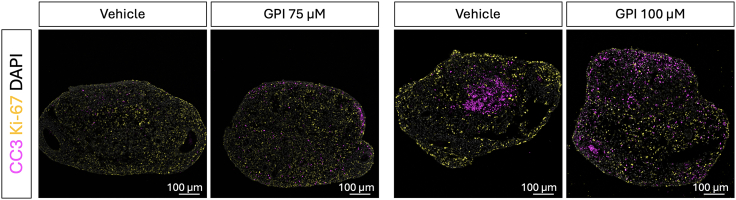
Representative immunofluorescence staining for proliferation and apoptosis markers in cryosections of vehicle or GPI treated gonads

### Whole-mount immunofluorescence

**Timing: 5 days**

This section describes how to process the gonadal samples for whole mount immunostaining.

Once a concentration of the inhibitor or treatment is selected, based on the previous histological analysis, we suggest performing whole mount immunofluorescence to properly quantify the changes in cellular numbers and 3D architecture.

44. Whole mount immunostaining.
  a. Transfer gonads to individual 0.5 Eppendorf tubes with 4:1 methanol:DMSO and store at −20 °C for at least 24 h.
  b. Wash the samples with 50% methanol diluted in 1X PBS for 30 min at room temperature,
  c. Perform three one-hour washes with blocking solution (10% donkey serum and 1% triton in 1X PBS) at room temperature.
  d. Incubate samples overnight at 4°C in blocking solution containing primary antibodies at desired concentration.
  e. Wash the samples 3 times with blocking buffer for 1 hour each at 4°C.
  f. Incubate with secondary antibodies in blocking solution overnight at 4°C.
  g. Dehydrate samples by performing 1-hour washes in increasing methanol concentrations (25%, 50%, 75% with DAPI, and 100%).
  h. Clear samples with 1:2 benzyl alcohol:benzyl benzoate (BABB) for at least 24 h.
  i. Acquire Z-stacks of the samples using a confocal microscope.

***Note:*** As an example, we are showing whole mount immunofluorescence for DDX4 (germ cell marker) in XY gonads treated with 75 μM GPI or vehicle solution (Methods videos S4, S5, S6, and S7).

Methods video S4. Confocal z-stack optical sections of vehicle treated gonads stained for DDX4, related to step 44

Methods video S5. Confocal z-stack optical sections of GPI treated gonads stained for DDX4, related to step 44

Methods video S6. 3D projection of vehicle treated gonads stained for DDX4, related to step 44

Methods video S7. 3D projection of GPI treated gonads stained for DDX4, related to step 44

### Single-gonad low-input total RNA-seq

**Timing: 5 days**

This section describes how to process the gonadal samples for low input RNA-seq where each gonad is sequenced considering the parity information (both gonads, control and treated one coming from the same fetus).

45. RNA extraction and library preparation.
  a. On collection day transfer the plate to a bucket with ice.
  b. Transfer one gonad-mesonephros complex to an empty glass dish using a plastic Pasteur pipette, forming a droplet.
  c. Separate gonad from mesonephros with a pair 1 ml syringes with 30-gauge needles, under a dissection stereoscope.
  d. Transfer the gonads into 1.5 ml Eppendorf tubes in dry ice and store them at −80 °C till processing.
    ***Note:*** We recommend dissecting each control and treated gonad pair sequentially before moving on to the next pair, to reduce experimental variability and ensure consistent exposure to handling and environmental conditions.
  e. Extract RNA from paired samples (control and treated) using a low input RNA extraction kit. We used the Arcturus™ PicoPure™ RNA Isolation Kit.
  f. The day of RNA extraction, transfer the tubes from the −80 °C to an ice bucket and spin them down briefly to pellet the samples to the bottom of the tube.
  g. Add 100 μl of extraction buffer to each tube and homogenize with a microtube homogenizer.
    ***Note:*** We recommend homogenizing each control and treated pair before moving on to the next pair, to reduce experimental variability and ensure consistent exposure to handling and environmental conditions.
  h. Incubate the samples at 42°C for 30 min, then freeze them at −80°C for at least one hour.
  i. Precipitate the RNA with 100 μl of 70% ethanol and load them into a pre-conditioned column.
  j. Centrifuge and wash the samples.
  k. Perform DNA removal using the RNase-Free DNase Set as suggested by the protocol.
  l. Elute samples in 12 μl of elution buffer.
  m. Quantify the obtained RNA using Qubit™ RNA High Sensitivity kit.
  n. Assess RNA quality using High Sensitivity RNA ScreenTape assay, in a TapeStation (Agilent).
    ***Note:*** If one of the samples from a pair does not meet the quality threshold (RIN score 8-10) or the concentration levels are undetectable by Qubit, we do not recommend submitting the pair for sequencing. We recommend submitting at least 4 pairs per treatment/control.
  o. Make cDNA libraries. We used Tecan’s Ovation® RNA-Seq System V2 followed by Tecan’s Celero™ EZ DNA-Seq.
  p. Sequence the libraries as paired-end 151-mers on an Illumina NextSeq 500 instrument.
    ***Note:*** For an example on sequencing results, please refer to Estermann et al.^1^

### Lactate measurement

**Timing: 5 h**

This section describes how to quantify Intracellular (gonadal) or extracellular (culture media) lactate concentration using the Lactate-Glo Assay.

**Figure 5:**
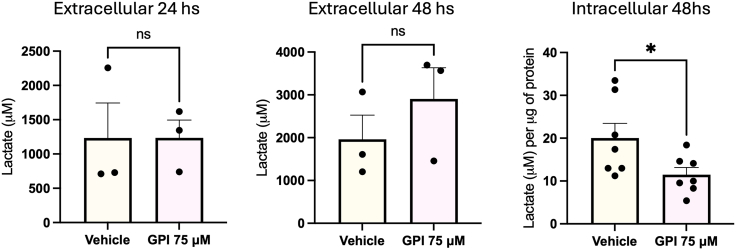
Representative measurements of extracellular and intracellular lactate levels in gonads cultured for 24 or 48 h Bars represent the mean ± SEM; Two-tailed *t* test. ∗*p* < 0.05; ns *p* > 0.05.

46. Sample processing:
  a. Intracellular lactate:
    i. On collection day transfer the plate to a bucket with ice.
    ii. Transfer one gonad-mesonephros complex to an empty glass dish using a plastic Pasteur pipette, forming a droplet.
    iii. Separate gonad from mesonephros with a pair 1 ml syringes with 30-gauge needles, under a dissection stereoscope.
    iv. Transfer the gonads into 1.5 ml Eppendorf tubes in dry ice and store them at −80 °C till processing.
      ***Note:*** We recommend dissecting each control and treated gonadal pair before moving on to the next pair, to reduce experimental variability and ensure consistent exposure to handling and environmental conditions.
    v. Transfer the tubes from the −80 °C to an ice bucket and spin them down briefly to pellet the samples to the bottom of the tube.
    vi. Homogenize each sample in 50 μl of homogenization buffer (50 mM Tris, pH 7.5) and 6.25 μl of inactivation solution (0.6 N HCl) using a microtube homogenizer.
      ***Note:*** We recommend homogenizing each control and treated gonadal pair before moving on to the next pair, to reduce experimental variability and ensure consistent exposure to handling and environmental conditions.
    vii. Add 6.25 μl of neutralization solution (1 M Tris base Trisma) to each sample immediately after homogenization.
    viii. Determine the protein concentration using 10 μl of the sample with the Qubit Protein Assay.
  b. Extracellular lactate:
    i. Dilute media collected at 24 or 48 h of culture 1/100 in sterile water.
47. Lactate detection:
  a. Add 50 μl of the processed samples in individual wells of a white 96 well plate.
    ***Note:*** A standard curve can be generated using serial dilution of lactate, starting at 200 μM, to interpolate lactate concentration from the samples. Process them as the samples.
  b. Add 50 μl of freshly prepared lactate detection reagent to each well and incubate for 60 min at room temperature.
  c. Record luminescence using the Promega GloMax Discoverer microplate reader.
  d. Determine the lactate concentration per sample by interpolation in the standard curve. For intracellular lactate, normalize lactate concentration to protein content.
    ***Note:*** As an example, we are showing gonadal intracellular and extracellular lactate levels of XY gonads treated with 75 μM GPI or vehicle solution for 24 or 48 h (Figure 5). Data visualization and statistical analysis (two-tailed t-test) was performed using GraphPad Prism.

## Expected outcomes

This protocol provides a comprehensive framework for assessing how different ex vivo culture conditions, including exposure to metabolic inhibitors, affect gonadal sex differentiation and development. Using the glycogen phosphorylase inhibitor (GPI) as an example, we first established the optimal working concentration (75 μM) that produced a phenotype without causing overwhelming cell toxicity or apoptosis. This was determined by measuring apoptosis and proliferation rates across a range of concentrations (Figure 4).

After defining the appropriate concentration, we performed multiple downstream characterizations to investigate the role of glycogen degradation in gonadal differentiation. Whole-mount immunofluorescence analysis revealed a 91% reduction in germ cell number upon inhibition of glycogen phosphorylation (Methods videos S4, S5, S6, and S7). To gain molecular insight into these phenotypes, we developed a paired single-gonad bulk RNA-seq, which identified 3,202 differentially expressed genes in response to glycogen degradation inhibition.^1^

Finally, because transcriptional data suggested that impaired glycogen degradation may lower lactate availability and thereby promote germ cell death, we measured both extracellular and intracellular lactate levels. Consistent with this hypothesis, intracellular gonadal lactate was significantly reduced, while extracellular lactate levels remained unchanged (Figure 5).

Together, these results illustrate the range of readouts that can be obtained using this protocol and highlight its utility for assessing the molecular and metabolic regulation of gonadal cell differentiation. For a full discussion of the outcomes and their biological implications, refer to Estermann et al.^1^

## Limitations

As with any ex vivo culture system, this protocol preserves gonadal identity but does not fully recapitulate the native three-dimensional architecture of the gonad in vivo. While overall tissue organization is largely maintained during short-term culture, modest architectural changes may occur over time and should be considered when interpreting results. Importantly, the paired-gonad design enables direct comparison, allowing treatment-induced effects to be distinguished from culture-associated changes. This protocol has been validated for mouse gonadal cultures at E11.5 and E12.5. Although application to later developmental stages may be feasible, increased tissue size may limit oxygen and nutrient diffusion, potentially leading to hypoxic regions and increased apoptosis within the tissue core. In addition, this methodology has not yet been tested in other species. Adaptation to additional developmental stages or model organisms will require further optimization and validation.

## Troubleshooting

### Problem 1

I have a different printer system; how can I be sure it is compatible?

### Potential solution

Because different printers and resins may introduce small differences in resolution, shrinkage, and surface finish, we recommend validating molds based on functional performance rather than strict dimensional tolerances. The mold does not need to be perfectly identical, but the final agar slab must fit inside a 48-well plate, hold 90 μl agar and form a dome, and generate a groove large enough to stabilize one fetal gonad without compressing it.

### Problem 2

Agar does not form a dome and runs down the resin mold.

### Potential solution

If the agar escapes the resin mold and fails to form a dome, allow the solution to cool for a few seconds and try again. Agar that is too hot remains too fluid to hold its shape.

### Problem 3

Gonad breaks during isolation or transfer to the agar slabs.

### Potential solution

If gonads are broken or not intact, we recommend discarding them, as tissue integrity is important for maintaining proper 3D architecture during culture.

### Problem 4

Gonad/mesonephros complexes are not as clean as in the dissection video.

### Potential solution

Excessive cleaning increases the risk of tissue damage. With practice, dissection quality will improve. We found that leaving small amounts of connective tissue does not negatively affect the culture system.

### Problem 5

Cellular debris appears when collecting gonads or changing media (including in control wells).

### Potential solution

This is normal, especially when residual connective tissue is present. Mesonephric tissue can also shed cells during culture.

### Problem 6

After clearing the samples in the last step of whole mount immunofluorescence, gonads are difficult to see and transfer to the microscope.

### Potential solution

This is why we include DAPI staining in the whole mount immunofluorescence protocol. Illuminating the samples with a handheld UV (black light) flashlight makes the gonads visible and facilitates transfer.

## Resource availability

### Lead contact

Requests for resources and reagents should be directed to the lead contact, Humphrey Hung-Chang Yao (humphrey.yao@nih.gov).

### Technical contact

Technical questions on executing this protocol should be directed to the technical contact, Martín Andrés Estermann (martin.estermann@nih.gov).

### Materials availability

This study did not generate new unique reagents.

### Data and code availability

This study did not generate new data or code.

## Acknowledgments

We would like to thank Barbara Nicol for her support in the production of the dissecting video and the sex genotyping protocol. This work was supported by the Intramural Research Program (Z01-ES102965 to H.H.-C.Y.) of the NIH, National Institute of Environmental Health Sciences. The contributions of the NIH authors were made as part of their official duties as NIH federal employees, are in compliance with agency policy requirements, and are considered works of the United States Government. However, the findings and conclusions presented in this paper are those of the authors and do not necessarily reflect the views of the NIH or the U.S. Department of Health and Human Services.

## Author contributions

Conceptualization, M.A.E.; methodology, M.A.E., K.F.R., and J.H.; writing – original draft, M.A.E. and J.H.; writing – review and editing, M.A.E., K.F.R., J.H., and H.H.-C.Y.; funding acquisition, H.H.-C.Y. All authors have read and agreed to the published version of the manuscript.

## Declaration of interests

The authors declare no competing interests.

## Supplemental information

File S1. 3D mold model for resin printing

## References

[1] Estermann M.A., Sheheen J., Grimm S.A., Tezak B., Chen Y.Y., Morita T., H-C Yao H., Capel B. (2026). Glycogen and lactate metabolism in mouse fetal Sertoli cells sustain the germ line. Cell Rep..

[2] Capel B., Batchvarov J. (2008). Preparing Recombinant Gonad Organ Cultures. CSH Protoc.

[3] Rudigier L.J., Dame C., Scholz H., Kirschner K.M. (2017). Ex vivo cultures combined with vivo-morpholino induced gene knockdown provide a system to assess the role of WT1 and GATA4 during gonad differentiation. PLoS One.

[4] Whiteley S.L., Holleley C.E., Georges A. (2025). In vitro organ culture protocol for intact urogenital systems supporting gonadal differentiation. PLoS One.

[5] Potter S.J., DeFalco T. (2015). Using Ex Vivo Upright Droplet Cultures of Whole Fetal Organs to Study Developmental Processes during Mouse Organogenesis. J. Vis. Exp..

[6] Schindelin J., Arganda-Carreras I., Frise E., Kaynig V., Longair M., Pietzsch T., Preibisch S., Rueden C., Saalfeld S., Schmid B. (2012). Fiji: an open-source platform for biological-image analysis. Nat. Methods.

[7] McFarlane L., Truong V., Palmer J.S., Wilhelm D. (2013). Novel PCR assay for determining the genetic sex of mice. Sex. Dev..

